# Combined rapamycin and mesenchymal stem/stromal cells derived from induced pluripotent stem cells-mediated delivery of ACVR2B-Fc fusion protein reduces heterotopic ossification in a mouse model of fibrodysplasia ossificans progressiva

**DOI:** 10.1093/jbmrpl/ziaf068

**Published:** 2025-04-21

**Authors:** Pan Gao, Yoshiko Inada, Maria José López-Iniesta, Chengzhu Zhao, Megumi Goto, Akitsu Hotta, Hidetoshi Sakurai, Makoto Ikeya

**Affiliations:** State Key Laboratory of Oral Diseases and National Center for Stomatology and National Clinical Research Center for Oral Diseases and Department of General Dentistry, West China Hospital of Stomatology, Sichuan University, Chengdu, Sichuan 610041, China; Department of Clinical Application, Center for iPS Cell Research and Application, Kyoto University, Kyoto 606-8507, Japan; Department of Clinical Application, Center for iPS Cell Research and Application, Kyoto University, Kyoto 606-8507, Japan; Department of Clinical Application, Center for iPS Cell Research and Application, Kyoto University, Kyoto 606-8507, Japan; Department of Clinical Application, Center for iPS Cell Research and Application, Kyoto University, Kyoto 606-8507, Japan; Laboratory of Skeletal Development and Regeneration, Key Laboratory of Clinical Laboratory Diagnostics (Ministry of Education), College of Laboratory Medicine, Chongqing Medical University, Chongqing 400016, China; Department of Clinical Application, Center for iPS Cell Research and Application, Kyoto University, Kyoto 606-8507, Japan; Department of Clinical Application, Center for iPS Cell Research and Application, Kyoto University, Kyoto 606-8507, Japan; Department of Clinical Application, Center for iPS Cell Research and Application, Kyoto University, Kyoto 606-8507, Japan; Department of Clinical Application, Center for iPS Cell Research and Application, Kyoto University, Kyoto 606-8507, Japan

**Keywords:** fibrodysplasia ossificans progressiva, heterotopic ossification, mesenchymal stem/stromal cells, ACVR2B-Fc fusion protein, rapamycin

## Abstract

Fibrodysplasia ossificans progressiva (FOP) is a genetic disease characterized by extraskeletal heterotopic ossification (HO). The underlying mechanism is the aberrant activation of bone morphogenetic protein (BMP) signaling caused by a point mutation in the *ACVR1* gene. Although FOP is an ultra-rare disease, its symptoms severely impair patients’ daily activities and quality of life. Furthermore, the HO is also observed in broader clinical contexts, including major surgeries and trauma, highlighting the significance of its study. We have previously reported that rapamycin suppressed Activin-A-triggered progression of cartilage formation in FOP mice. We recently found that the ACVR2B-Fc fusion protein produced by mesenchymal stem/stromal cells derived from induced pluripotent stem cells (iMSCs) attenuates BMP signaling activated by Activin-A and BMP-9 in FOP patient-derived iMSCs. Transplantation of ACVR2B-Fc-producing iMSCs (iMSC^2B-Fc/Luci^) reduces primary HO in FOP mice. In this study, we found that intraperitoneal administration of rapamycin reduced primary HO in a dose-dependent manner in FOP-ACVR1^R206H^ transgenic mice. A low concentration of rapamycin (0.3 mg/kg, 5 times/wk) efficiently suppressed chondrogenesis. The combination of iMSC^2B-Fc/Luci^ transplantation with low concentration of rapamycin significantly reduced primary and recurrent HO. Rapamycin improved cell survival in transplanted iMSC^2B-Fc/Luci^ cells by attenuating chemokine secretion, likely resulting in improved ACVR2B-Fc fusion protein production. Our results suggest that combining stem cell therapy and rapamycin can reduce primary HO and surgery-induced HO.

## Graphical Abstract

**Graphical Abstract:**
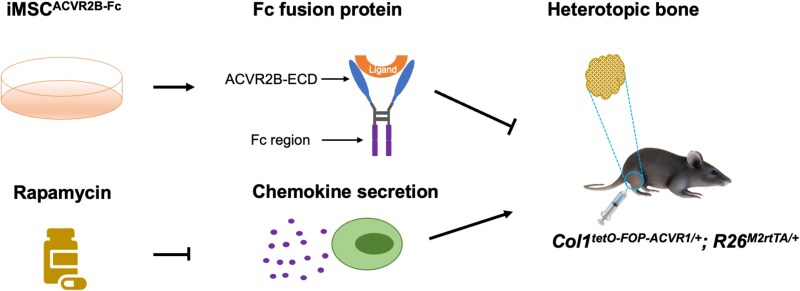

## Introduction

Fibrodysplasia ossificans progressiva (FOP) is caused by a gain-of-function mutation in the glycine- and serine-rich (GS) domain of the *ACVR1* gene, which results in extraskeletal heterotopic ossification (HO).^1^ FOP flare-ups arise spontaneously or are triggered by soft tissue injuries caused by accidental trauma, muscular stretching, fatigue, influenza-like illness, intramuscular vaccination, biopsies, and surgeries.^2^ Thus, surgical resection of HO lesions in patients with FOP is prohibited as the resulting injury causes local and systematic aggravation of HO. Drug candidates of FOP can be classified into three categories based on their blocking sites: (1) extracellular antibodies or receptobodies targeting activins, bone morphogenetic proteins (BMPs), or the extracellular domain of ACVR1; (2) intracellular kinase inhibitors targeting the kinase domain of ACVR1; and (3) intracellular agonists or inhibitors targeting downstream effectors.

As a molecular inhibitor of downstream, rapamycin reportedly suppressed trauma-induced and genetic (*ACVR1^Q207D^*) de novo HO by inhibiting Hif1𝑎.^3^ The same group also found that rapamycin reduces fibrosis and mesenchymal condensation at the muscle injury site, which results in heterotopic osseous lesions.^4^ We also identified the mammalian target of rapamycin (mTOR) signaling as a crucial pathway for the abnormal chondrogenesis of mesenchymal stromal/stem cells derived from FOP-patient induced pluripotent stem cells (FOP-iMSCs) using high-throughput screening.^5^ Oxidative phosphorylation is a downstream mediator in mTORC1-induced chondrogenesis.^6^ Oral administration of rapamycin (3, 10, and 30 mg/kg; 5 times/wk) dose-dependently suppresses BMP-7-induced HO in C57BL/6 mice and FOP-iMSCs-derived HO in NOD/SCID mice, wherein 10 mg/kg of rapamycin inhibits > 95% of HO compared with the control.^5^ Intraperitoneal administration of rapamycin (5.0 mg/kg, 5 times/wk) suppresses primary HO and surgery-induced HO in FOP mice.^7^ When converting the above-mentioned effective dose for mouse to human using the equation of human equivalent dose (HED) (mg/kg) = animal no observed adverse effect levels (NOAEL) (mg/kg) × (Weight_animal_ [kg]/Weight_human_ [kg])^(1-0.67)^ or HED (mg/kg) = Animal dose (mg/kg) × (Animal *K_m_*/Human *K_m_*),^8^ the effective rapamycin dose for human will be as much as 10 times higher than the maintenance dose for prophylaxis of renal transplant rejection (PO, 2 mg per day)^9^ and the current clinical trial to reduce FOP-HO (PO, 1.0-4.0 mg per day, UMIN000028429). Thus, it is necessary to determine the minimal effective concentration of rapamycin required to suppress HO in a mouse model.

Besides, extracellular recombinant antibody-like ACVR2A-Fc and ACVR2B-Fc profoundly inhibit Activin-A-dependent BMP signaling and chondrogenesis in vitro^10^ and prevent HO formation in vivo.^11^ However, commercial ACVR2A-Fc and ACVR2B-Fc are extremely expensive. We have recently reported that iMSC-mediated delivery of the ACVR2B-Fc fusion protein reduced primary HO in FOP mice, suggesting a novel approach for treating FOP through stem cell therapy.^12^ However, short-term survival after iMSC^ACVR2B-Fc^ transplantation and the limited reduction of recurrent HO necessitate optimization of the iMSC^ACVR2B-Fc^ delivery strategy. In this study, we investigated the immunomodulatory effects of rapamycin beyond its ability to suppress HO, enhancing the performance of transplanted iMSC^ACVR2B-Fc^ in reducing primary and recurrent HO.

## Materials and methods

### Mouse model and in-vivo imaging

Animal procedures were approved by the Institutional Animal Committee of Kyoto University (#16-73, #22-154, #22-155, and #23-218). The FOP-ACVR1^R206H^ transgenic mouse model has been described^12^ with minor modifications. Sample size of mice was calculated based on the reported formula.^13^ Four to five female mice aged 16-21 wk were randomly allocated into each group using a random-number table. Rapamycin (MedChemExpress, dissolved in 10% DMSO with 0.5% w/v methylcellulose 400 solution) at gradient concentrations (0, 0.1, 0.3, 0.6, 2.5, and 5.0 mg/kg; 5 times/wk) was intraperitoneally administered as the injury of gastrocnemius muscle was induced by cardiotoxin (CTX, 9.1 μg/mouse, MedChemExpress). Three injections of 1.5 × 10^6^ cells in 100 μL Alpha Modification of Eagle's Medium (aMEM) were locally administered every 6-7 d using a 27G syringe in the primary HO model and two injections for the recurrent HO model. X-ray and μCT imaging and calculation of the volume of heterotopic bone were performed as previously described. A 13-wk-old NOD-SCID IL2Rγc^null^ (NSG) mice (Jackson Laboratory) was used for transplanting cells (iMSC^EiP/Luci^, 3 × 10^6^) through the right gastrocnemius muscle, IP, and tail vein injection. Luminescent signals were detected using an in-vivo imaging system (PerkinElmer). The mice that died before the endpoint of the experiments were excluded from the analysis.

### Cell culture

The induction and maintenance of iPSC-derived neural crest cells (iNCCs) and MSCs (iMSCs) were described previously.^14^ The generation of iMSC^2B-Fc/Luci^ and iMSC^EiP/Luci^ cells were performed as previously described.^12^ Briefly, 5 μg of pPV-EF1a-Luciferase-BleoR and 1 μg of PBase plasmids were respectively mixed with 5 μg of pPB-CAG-ACVR2B-Fc-His-Puro or 5 μg of pPV-EF1a-EGFP-IRES-Puro. A total of 100 μL of the DNA and iMSC mixture was obtained. After electroporation using a NEPA21 electroporator (Nepagene, Japan), stably expressing cells were screened using 1 μg/mL puromycin (Gibco) with 400 μg/mL zeocin (InvivoGen) for 7 d.

### Rotarod and treadmill test

Rotarod and treadmill tests were referred previously.^12^ Briefly, mice were loaded onto a rotarod station (ENV-574M, Med Associates) and Rodent Treadmill (Ugo Basile SRL). The rotation speed was set from 2 to 20 rpm and maintained at 20 rpm until the mice fell. The riding times were recorded. The treadmill movement test (+5° inclination) started at a 5 m/min velocity, gradually increasing to a maximum of 20 m/min (shock: 1 Hz, shock intensity 0.2 mA). Exhaustion was defined as the mice being shocked 15 times or not returning to the treadmill and staying on the shock grid for 5 s. Running distances were recorded.

### ELISA

Mouse blood samples were left at room temperature (RT, 20-25 °C) for 30 min until clot formation. The serum was harvested after centrifugation for 15 min at 2000 × *g*. Activin-A and Fc fragment in serum were detected using Human/Mouse/Rat Activin-A Quantikine ELISA Kit (DAC00B, R&D Systems) and Human Fcγ (Fc Fragment of IgG) ELISA Kit (E-EL-H1745, Elabscience). The absorbances at 450 nm were read using an EnVision Multilabel Reader (PerkinElmer).

### Cytokine array

The serum (150 μL) of each mouse was then subjected to cytokine array analysis (Mouse Cytokine Array Panel A, R&D Systems, ARY006) according to the manufacturer’s instructions. Spot pixel density was quantified using the Protein Array Analyzer of ImageJ software (https://imagej.net/ij/).

### Histochemical and IHC analyses

Fixed and decalcified samples (4% paraformaldehyde for 24 hr, 12% EDTA for 10 d) were embedded in paraffin, sectioned, deparaffinized and stained with H&E and Alcian blue. For IHC staining, antigen retrieval was performed via incubation with Liberate Antibody Binding Solution (Polysciences, USA) for 20 min at RT. Non-specific antigens were blocked using Blocking One (Nacalai Tesque) for 1 hr at RT. For antibodies generated from the mouse host, tissues were blocked with ReadyProbes Mouse on Mouse IgG Blocking Solution (Invitrogen) for 1 hr at RT. Antibodies (diluted in Can Get Signal Immunostain Solution B, TOYOBO) were incubated overnight at 4 °C. After rinsing in PBST buffer, samples were incubated with Alexa Fluor secondary antibodies diluted in Can Get Signal Immunostain Solution B for 1 hr at RT. A mounting medium with DAPI (Vector Laboratories) was used to counterstain the nuclei. Images were captured using a BZ-X810 microscopy (Keyence). The antibodies for IHC are listed in Table 1.

**Table 1:** Immunohistochemical antibodies.

**Name**	**Company**	**Cat. No.**	**Concentration**
**Collagen I alpha 1 antibody (COL-1)**	Novus Biologicals	NB600-450	1:100
**Anti-human vimentin antibody**	Abcam	ab16700	1:100
**Donkey anti-rabbit IgG (H + L), Alexa Fluor 488**	Invitrogen	A21206	1:100
**Goat anti-mouse IgG (H + L), Alexa Fluor 555**	Invitrogen	A21422	1:100

### Statistical analysis

The statistical significance of all experiments was calculated using one-way analysis of variance with Dunnett’s or Tukey’s multiple comparison test and two-way analysis of variance with Tukey’s multiple comparisons test, as indicated in each figure legend. All statistical analyses were performed using GraphPad Prism version 9.5.1 (GraphPad Software). A *p*-value <.05 were considered statistically significant. Significance levels are: ^*^p < .05; ^**^p < .01; ^***^p < .001; ^****^p < .0001.

## Results

### Intraperitoneally administered rapamycin reduced primary HO in a dose-dependent manner

**Figure 1:**
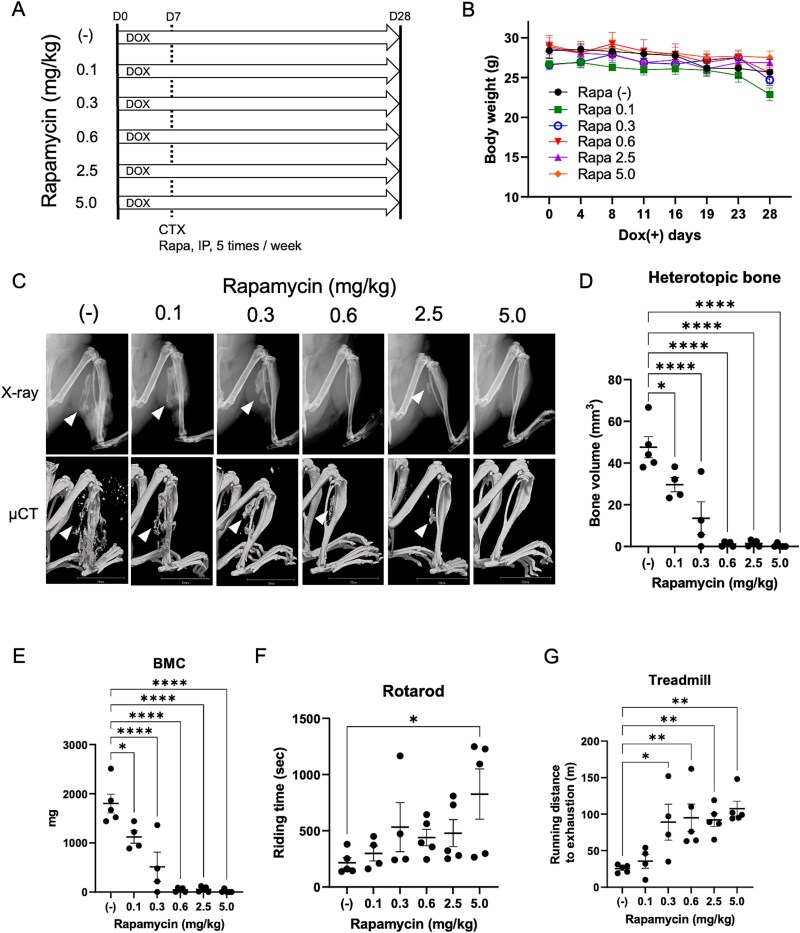
Intraperitoneally administered rapamycin reduces primary HO in a dose-dependent manner. (A) Schematic view of the experiments. Female mice aged 16 wk were administrated with drinking water supplemented with 2 mg/mL DOX and 10 mg/mL sucrose to initiate the FOP-ACVR1^R206H^ expression. CTX (9.1 μg/mouse) was injected in gastrocnemius on day 7. Rapamycin with indicated concentrations (0, 0.1, 0.3, 0.6, 2.5, 5.0 mg/kg) was intraperitoneally administrated from day 7 (5 times/wk). (B) The body weight of mice was monitored every 3-5 d. (C) Representative X-ray and μCT images from each group. Arrowheads indicate HO. Scale bar, 10 mm. (D) Average of HO volume on day 28. (E) BMC of HO lesions. (F) The exercise capacities were analyzed using the rotarod test. (G) Endurance tests were evaluated using a rodent treadmill. Results represent the mean ± SEM. ^*^, *p* < .05; ^**^, *p* < .01; ^****^, *p* < .0001. Data were analyzed using one-way ANOVA with Dunnett’s multiple comparison test. Abbreviations: HO, heterotopic ossification; DOX, doxycycline hyclate; CTX, cardiotoxin; BMC, bone mineral content.

Rapamycin (0, 0.1, 0.3, 0.6, 2.5, and 5.0 mg/kg; i.p., 5 times/wk) was administered on the same day that the muscle injury was triggered by CTX injection (Figure 1A). The body weights of the mice in most of the groups remained relatively stable throughout the experiments (Figure 1B). At the end of the experiment, mice were subjected to X-ray and μCT scans under general anesthesia (Figure 1C). Quantitative bone volume analysis revealed that rapamycin reduced primary HO levels in a dose-dependent manner. Rapamycin (0.3 mg/kg) inhibited HO by ⁓60% (Figure 1D). The bone mineral content (BMC) of HO was consistent with the bone volume (Figure 1E). Rapamycin also dose-dependently improved the rotarod (Figure 1F) and treadmill (Figure 1G) performance. Positive staining for Alcian blue (cartilage) and COL1 (bone) indicated the occurrence of endochondral ossification (Figure S1).

### Low-dose rapamycin enhanced the effects of iMSC ^ACVR2B-Fc^ to reduced primary HO

**Figure 2:**
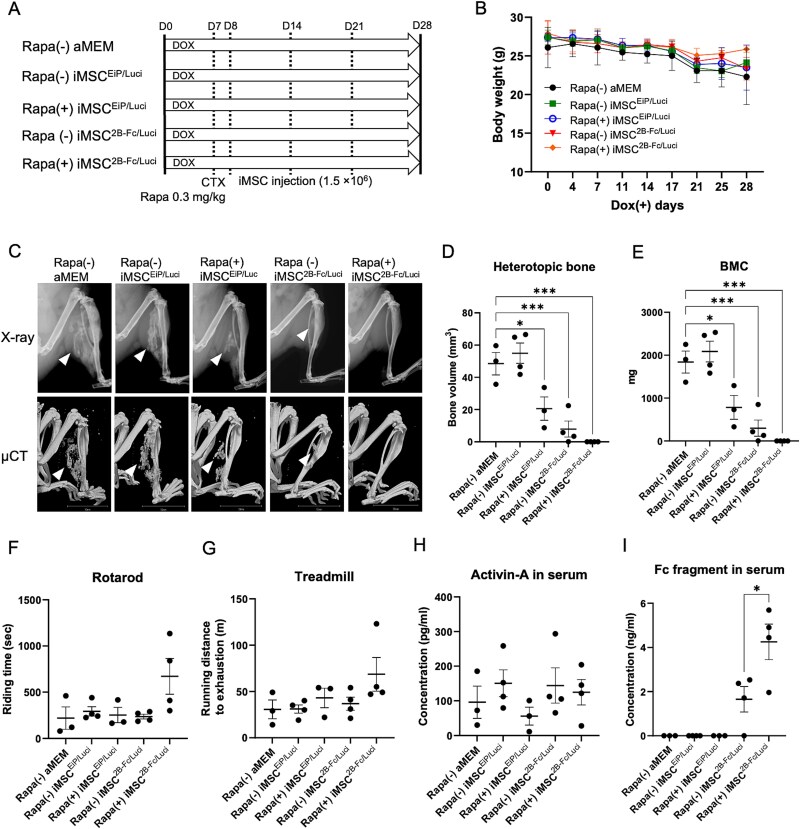
Low-dose rapamycin enhanced the effects of iMSC^2B-Fc/Luci^ to reduce primary HO. (A) Schematic view of the experiments. Rapamycin (0.3 mg/kg, 5 times/wk) was intraperitoneally administrated after CTX injected on day 7. Cells in 100 μL aMEM (iMSC^EiP/Luc^ or iMSC^2B-Fc/Luc^, 1.5 × 10^6^) were locally administered every 7 d from day 8. (B) The body weight of mice was monitored every 3-5 d. (C) Representative X-ray and μCT images from each group. Arrowheads indicate HO. Scale bar, 10 mm. (D) Average of HO volume on day 28. (E) BMC of HO lesions. (F) The exercise capacities were analyzed using the rotarod test. (G) Endurance tests were evaluated using a rodent treadmill. (H, I) Activin-A and Fc-fragment in mouse serum were detected by ELISA. Results represent the mean ± SEM. ^*^, *p* < .05; ^**^, *p* < .01; ^***^, *p* < .001. Data were analyzed using one-way ANOVA with Dunnett’s multiple comparison test. Data in (I) were analyzed using one-way ANOVA with Tukey’s multiple comparison test. Abbreviations: HO, heterotopic ossification; iMSC, mesenchymal stem/stromal cells derived from induced pluripotent stem cells; CTX, cardiotoxin; aMEM, Alpha Modification of Eagle's Medium; BMC, bone mineral content.

Luciferase-expressing iMSC^EiP/Luci^ and iMSC^2B-Fc/Luci^ cells were generated as previously described.^12^ In-vivo imaging was performed in the NSG and FOP mice to determine the survival time of the transplanted cells under immunodeficient and immunocompetent conditions. iMSC^EiP/Luci^ cells (3 × 10^6^) resuspended in 100 μL aMEM medium were transplanted into NSG mice through the right gastrocnemius muscle, IP, and tail vein injection. Only a weak luciferase signal was detected in the systematically delivered (tail vein) mouse on day 1 after transplantation (Figure S2A). The luciferase signal rapidly decreased and was undetectable on day 7. As muscle injury was triggered by CTX, rapamycin (0.3 mg/kg, 5 times/wk) was administered intraperitoneally on day 7. iMSC^EiP/Luci^ or iMSC^2B-Fc/Luci^ cells (1.5 × 10^6^) were locally injected into the injured muscle from day 8 (Figure 2A). The luciferase signals in the mice treated with rapamycin decreased more slowly over time than those in the control and were detectable for at least 5-6 d, although the differences were not statistically significant (Figure S2B). Thus, three injections of 1.5 × 10^6^ cells were locally administered every week (Figure 2A). The weights of the mice in all groups were comparable and slightly decreased over time (Figure 2B). The X-ray and μCT scans showed that low rapamycin concentrations combined with iMSC^2B-Fc/Luci^ significantly suppressed primary HO, wherein no HO was formed in two out of four mice (Figure 2C-E). This combination improved the rotarod and treadmill performance to some extent, although no statistical difference was observed (Figure 2F and G). No significant difference was found in serum Activin-A levels among the groups (Figure 2H). Rapamycin increased the secretion of Fc fusion proteins in the serum (Figure 2I), suggesting that it increased the survival or function of transplanted iMSC^2B-Fc/Luci^ cells. H&E staining of the whole section showed that the combined administration decreased the area of the HO lesions (Figure S3). The transplanted iMSCs were marked by vimentin, which did not co-stain with the mouse-derived COL1^+^ bone tissue, suggesting that the transplanted iMSCs did not differentiate into bone tissue.

### Low-dose rapamycin enhanced the effects of iMSC ^2B-Fc/Luci^ to reduced recurrent HO

**Figure 3:**
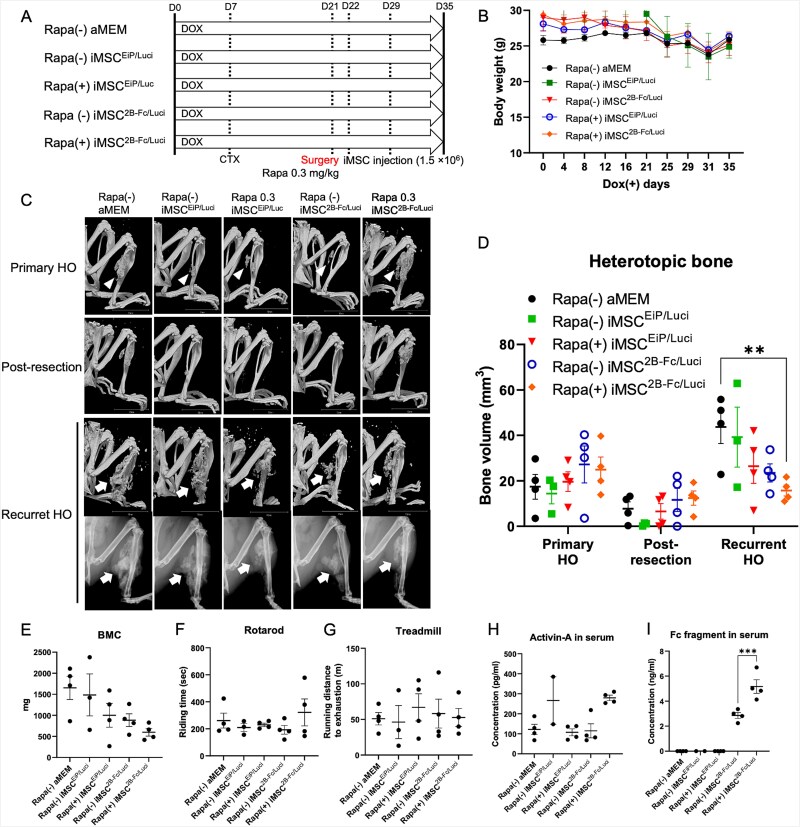
Low-dose rapamycin enhanced the effects of iMSC^2B-Fc/Luci^ to reduce recurrent HO. (A) Schematic view of the experiments. Rapamycin (0.3 mg/kg, 5 times/wk) was intraperitoneally administrated after HO was surgically removed on day 21. Cells in 100 μL aMEM (iMSC^EiP/Luc^ or iMSC^2B-Fc/Luc^, 1.5 × 10^6^) were locally administered every 7 d from day 22. (B) The body weight of mice was monitored every 3-5 d. (C) Representative X-ray and μCT images from each group. Arrowheads indicate primary HO, and arrows indicate recurrent HO. Scale bar, 10 mm. (D) Average of HO volume on day 21 (pre- and post-surgery) and recurrent HO (volume_day35_ – volume_post-surgery_). (E) BMC of HO lesions. (F, G) The exercise capacity and endurance were evaluated using the rodent rotarod and treadmill tests, respectively. (H, I) Activin-A and Fc-fragment in mouse serum were detected by ELISA. Results represent the mean ± SEM. ^**^, *p* < .01; ^***^, *p* < .001. Data were analyzed using one-way ANOVA with Dunnett’s multiple comparison test. Data in (D) were analyzed using two-way ANOVA with Tukey’s multiple comparison test. Data in (I) were analyzed using one-way ANOVA with Tukey’s multiple comparison test. Abbreviations: HO, heterotopic ossification; iMSC, mesenchymal stem/stromal cells derived from induced pluripotent stem cells.

On day 21, as many HO lesions as possible were surgically resected under general anesthesia (Figure 3A). Rapamycin (0.3 mg/kg, 5 times/wk) was intraperitoneally administered. iMSC^EiP/Luci^ or iMSC^2B-Fc/Luci^ cells (1.5 × 10^6^) were locally transplanted every week from day 22. The weights of mice in all groups decreased after surgery but were comparable with time (Figure 3B). X-ray and μCT scans were performed on days 21 (pre- and post-resection) and 35, respectively (Figure 3C). The volume of recurrent HO was the volume on day 35 subtracted from the remaining post-resection volume on day 21. Rapamycin in combination with iMSC^2B-Fc/Luci^ significantly reduced the recurrent HO (Figure 3D) and BMC (Figure 3E). No statistical differences were observed in rotarod and treadmill performance (Figure 3F and G) or Activin-A levels in mouse serum (Figure 3H). Increased secretion of the Fc fusion protein into the serum was also confirmed in the combination group (Figure 3I). Histological and immunohistochemical (IHC) analyses of HO lesions were similar to those described above (Figure S4).

### Rapamycin attenuated cytokine secretion initiated by iMSC transplantation

**Figure 4:**
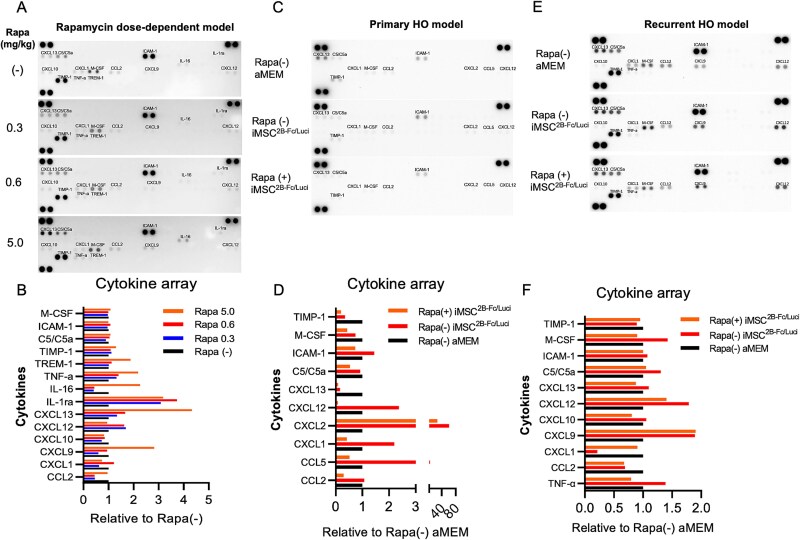
Rapamycin attenuated secretion of cytokines initiated by iMSC transplantation. (A) The mouse serum in the rapamycin dose-dependent model (0, 0.3, 0.6, 5.0 mg/kg) was subjected to cytokine array assay. The spots on the lower right corner of the membrane (invisible) represent negative control. The spots on the other three corners indicate positive control. (B) The pixel density of the targets was normalized to the pixel density of the positive control on the upper left corner of the membrane. Quantifications are relative to the group of rapamycin (−). (C, E) Mouse serum from the indicated groups in both primary (C) and recurrent (E) HO models was subjected to cytokine array assay. (D, F) Quantifications are relative to the group of rapamycin (−) and aMEM. Abbreviations: HO, heterotopic ossification; iMSC, mesenchymal stem/stromal cells derived from induced pluripotent stem cells.

Cytokine array assays were conducted to examine cytokine secretion in FOP mice under different treatments. In the rapamycin dose-dependent model, the low concentration of rapamycin (0.3 mg/kg) was likely to reduce cytokines, including chemokines CCL2, CXCL1, CXCL9, and CXCL10, and pro-inflammatory IL-16 (Figure 4A and B). In the primary HO model, rapamycin (0.3 mg/kg) was likely to decrease the increase in cytokines triggered by iMSC^2B-Fc/Luci^ transplantation, including the chemokines CCL2, CCL5, CXCL1, CXCL2, and CXCL12, and the soluble cell adhesion molecule ICAM-1 (Figure 4C and D). In the recurrent HO model, rapamycin decreased the enhancement of cytokines triggered by iMSC^2B-Fc/Luci^ transplantation, including pro-inflammatory TNF-𝑎, chemokines CXCL10, CXCL12, and CXCL13, anaphylatoxin C5/C5a, soluble cell adhesion molecule ICAM-1, and hematopoietic growth factor M-CSF (Figure 4E and F).

## Discussion

We recently developed xeno-free iMSCs derived from induced pluripotent stem cells (iPSCs), which resemble the tissue-derived MSCs and promote skeletal muscle regeneration via secretion of soluble factors.^14^ Although the muscle injury was induced via CTX injection or surgery in the FOP, the transplantation of control iMSCs (iMSC^EiP/Luci^) did not inhibit primary or recurrent HO (Figures 2D and 3D). In contrast, transplantation of ACVR2B-Fc-producing iMSCs (iMSC^2B-Fc/Luci^) reduced primary HO and recurrent HO, suggesting that the aberrant activation of BMP signaling caused by the FOP-ACVR1 mutant is hypersensitive and rapidly responsive to injury, which cannot be mitigated by transplanted iMSC^EiP/Luci^.

MSCs express low levels of major histocompatibility complexes (MHC) and activate natural killer (NK) cell receptors on their surfaces upon interaction with immune cells.^15^ However, interferon-gamma is well-documented to enhance MHC class I levels and triggers the expression of MHC class II in MSCs, potentially increasing their immunogenicity and leading to a detrimental effect on allograft survival.^16^^,^^17^ Thus, MSCs are regarded as immune evasive rather than immune privileged.^18^ Consequently, MSCs are susceptible to be attacked by cytotoxic CD8^+^ T cells and NK cells.^19^ Thus, multiple transplantations of MSCs must be conducted, which increases costs and hampers clinical application.^20^ Priming MSCs with inflammatory cytokines typically results in the temporary upregulation of different immunosuppressive molecules and improves the survival of transplanted cell grafts.^21–23^ Additionally, the combination of rapamycin (an immunosuppressant) and MSCs fostered immunotolerance in animal models of pancreatic islet^24^ and heart transplantation.^25^

In our previous study, the host immune rejection and a surgery-induced cytokine storm attenuated the survival and capability of xenogenic iMSCs to produce the ACVR2B-Fc fusion protein.^12^ Thus, we hypothesized that rapamycin benefits the transplantation of iMSC^2B-Fc/Luci^ by prolonging survival duration, reducing management frequency, and inhibiting HO efficiently. Even in the immunodeficient NSG mice, most transplanted iMSCs survived for under 7 d (Figure S2A). Rapamycin administration (0.3 mg/kg) was likely to extend the survival time of transplanted iMSCs to most 6 d (Figure S2B). Rapamycin (0.3 mg/kg) combined with iMSC^ACVR2B-Fc^ significantly reduced primary (Figure 2D) and recurrent (Figure 3D) HO. Combined administration also improved rotarod and treadmill performances in the primary HO model, although the differences were not statistically significant (Figure 2F and G). In the recurrent HO, the HO volume data and the rotarod and treadmill performance data did not show consistent trends (Figure 3D-G), unlike in primary HO (Figure 2D-G), which showed more consistent trends. This may be due to the fact that in the FOP mice, to remove the primary HO lesion, a long muscle damage was formed very close to the knee joint, and therefore the recurrent HO often formed near the knee, resulting in ankylosis of the joint in all groups. Given that symptom relief and functional recovery are critical for patients, further research is needed to find ways to prevent re-ossification, especially near joints. Notably, rapamycin increased the secretion of the ACVR2B-Fc fusion protein in the serum (Figures 2I and 3I), suggesting that it promoted cell survival or improved the production function of transplanted iMSCs. Chemokines are chemotactic cytokines that play a crucial role in regulating the migration and localization of immune cells within tissues, and are essential for the function of the innate immune system. Moreover, they facilitate the release of innate immune cells from the bone marrow and their recruitment from the bloodstream to affected tissues in response to infection and inflammation. Additionally, chemokines are vital for positioning innate immune sentinels in peripheral tissues, and they guide these activated cells to the nearest lymph nodes to initiate and shape an adaptive immune response upon activation of the innate immune system.^26^ The cytokine array kit used in this current study (R&D Systems, ARY006) detected 40 cytokines, chemokines, and acute-phase proteins, including anti-inflammatory, pro-inflammatory, and immunomodulatory factors. A low rapamycin concentration mainly reduced immune activation by suppressing chemokine secretion upon local delivery of iMSC^2B-Fc/Luci^ (Figure 4D and F).

The clinical application of rapamycin has several disadvantages, including poor water solubility, first-pass metabolism, low oral bioavailability, and specific binding to hemoglobin.^27^ Recently, various formulation strategies (particularly the use of nanostructured carriers) were developed to overcome these disadvantages.^28^ Injectable suspensions of rapamycin albumin-bound particles are approved by the U.S. FDA for adult patients with locally advanced, unresectable, or metastatic malignant perivascular epithelioid cell tumors (www.fda.gov). To overcome poor water solubility, rapamycin was dissolved in 10% dimethyl sulfoxide (DMSO) with a 0.5% w/v methylcellulose 400 solution. Intraperitoneal administration of rapamycin dose-dependently reduced primary HO and improved rotarod and treadmill performances (Figure 1C-G). Even a low rapamycin concentration (0.3 mg/kg) reduced ⁓60% HO (Figure 1E). Recently, hybrid spheroids consisting of rapamycin-releasing microparticles and MSCs markedly extended the survival of islets and facilitated the production of regional T_reg_.^29^ This drug system realizes long-term immunosuppression via a single administration of low-dose rapamycin, which may reduce systemic immunodeficiency and toxic effects caused by long-term exposure to rapamycin.

In this study, human-derived iMSCs were used to deliver the therapeutic protein (ACVR2B-Fc) to the immunocompetent FOP mice. The human leukocyte antigen (HLA) carried by iMSCs may compromise their immunomodulation and tissue regeneration functions. iMSCs derived from HLA genome-edited iPSCs will further improve immune compatibility and may be an off-the-shelf product for clinical application.^30^ The fact that a low rapamycin concentration promoted cell survival and production of the ACVR2B-Fc fusion protein and synergistically reduced primary and recurrent HO indicated that the combination of stem cell therapy and immunosuppressants may benefit patients with FOP by reducing recurrent HO caused by surgical removal of the primary lesion. Therefore, surgical procedures for the removal of HO may be feasible. Furthermore, the hybrid spheroids consisting of rapamycin-releasing microparticles and MSCs reported by Nguyen et al. offer a promising strategy for optimizing this combination therapy for FOP.

## Supplementary Material

Figure_S1_ziaf068

Figure_S2_ziaf068

Figure_S3_ziaf068

Figure_S4_ziaf068

Figure_captions_ziaf068

## Data Availability

All data supporting the findings of this study are available within the paper and its Supplementary Information.

## References

[1] Shore EM, Xu M, Feldman GJ, et al. A recurrent mutation in the BMP type I receptor ACVR1 causes inherited and sporadic fibrodysplasia ossificans progressiva. Nat Genet. 2006;38(5):525–527. 10.1038/ng1783 16642017

[2] Ventura F, Williams E, Ikeya M, et al. Challenges and opportunities for drug repositioning in Fibrodysplasia Ossificans Progressiva. Biomedicines. 2021;9(2):213. 10.3390/biomedicines9020213 33669809 PMC7922784

[3] Agarwal S, Loder S, Brownley C, et al. Inhibition of Hif1α prevents both trauma-induced and genetic heterotopic ossification. Proc Natl Acad Sci USA. 2016;113(3):E338–E347. 10.1073/pnas.1515397113 26721400 PMC4725488

[4] Agarwal S, Cholok D, Loder S, et al. mTOR inhibition and BMP signaling act synergistically to reduce muscle fibrosis and improve myofiber regeneration. JCI Insight. 2016;1(20):e89805. 10.1172/jci.insight.89805 27942591 PMC5135269

[5] Hino K, Horigome K, Nishio M, et al. Activin-A enhances mTOR signaling to promote aberrant chondrogenesis in fibrodysplasia ossificans progressiva. J Clin Invest. 2017;127(9):3339–3352. 10.1172/JCI93521 28758906 PMC5669572

[6] Sun L, Jin Y, Nishio M, et al. Oxidative phosphorylation is a pivotal therapeutic target of fibrodysplasia ossificans progressiva. Life Sci Alliance. 2024;7(5):e202302219. 10.26508/lsa.202302219 38365425 PMC10875110

[7] Maekawa H, Kawai S, Nishio M, et al. Prophylactic treatment of rapamycin ameliorates naturally developing and episode -induced heterotopic ossification in mice expressing human mutant ACVR1. Orphanet J Rare Dis. 2020;15(1):122. 10.1186/s13023-020-01406-8 32448372 PMC7245788

[8] Nair AB, Jacob S. A simple practice guide for dose conversion between animals and human. J Basic Clin Pharm. 2016;7(2):27–31. 10.4103/0976-0105.177703 27057123 PMC4804402

[9] Vasquez EM . Sirolimus: a new agent for prevention of renal allograft rejection. Am J Health Syst Pharm. 2000;57(5):437–448. 10.1093/ajhp/57.5.437 10711524

[10] Hino K, Ikeya M, Horigome K, et al. Neofunction of ACVR1 in fibrodysplasia ossificans progressiva. Proc Natl Acad Sci USA. 2015;112(50):15438–15443. 10.1073/pnas.1510540112 26621707 PMC4687587

[11] Hatsell SJ, Idone V, Wolken DMA, et al. ACVR1R206H receptor mutation causes fibrodysplasia ossificans progressiva by imparting responsiveness to Activin A. Sci Transl Med. 2015;7(303):303ra137. 10.1126/scitranslmed.aac4358 PMC6164166 26333933

[12] Gao P, Inada Y, Hotta A, Sakurai H, Ikeya M. iMSC-mediated delivery of ACVR2B-Fc fusion protein reduces heterotopic ossification in a mouse model of fibrodysplasia ossificans progressiva. Stem Cell Res Ther. 2024;15(1):83. 10.1186/s13287-024-03691-7 38500216 PMC10949803

[13] Arifin WN, Zahiruddin WM. Sample size calculation in animal studies using resource equation approach. Malays J Med Sci MJMS. 2017;24(5):101–105. 10.21315/mjms2017.24.5.11 PMC5772820 29386977

[14] Kamiya D, Takenaka-Ninagawa N, Motoike S, et al. Induction of functional xeno-free MSCs from human iPSCs via a neural crest cell lineage. Npj Regen Med. 2022;7(1):1–17. 10.1038/s41536-022-00241-8 36109564 PMC9477888

[15] Schu S, Nosov M, O’Flynn L, et al. Immunogenicity of allogeneic mesenchymal stem cells. J Cell Mol Med. 2012;16(9):2094–2103. 10.1111/j.1582-4934.2011.01509.x 22151542 PMC3822979

[16] Krampera M, Glennie S, Dyson J, et al. Bone marrow mesenchymal stem cells inhibit the response of naive and memory antigen-specific T cells to their cognate peptide. Blood. 2003;101(9):3722–3729. 10.1182/blood-2002-07-2104 12506037

[17] Chan WK, Lau AS-Y, Li JC-B, Law HK-W, Lau YL, Chan GC-F. MHC expression kinetics and immunogenicity of mesenchymal stromal cells after short-term IFN-gamma challenge. Exp Hematol. 2008;36(11):1545–1555. 10.1016/j.exphem.2008.06.008 18715686

[18] Ankrum JA, Ong JF, Karp JM. Mesenchymal stem cells: immune evasive, not immune privileged. Nat Biotechnol. 2014;32(3):252–260. 10.1038/nbt.2816 24561556 PMC4320647

[19] Crop MJ, Korevaar SS, De Kuiper R, et al. Human mesenchymal stem cells are susceptible to lysis by CD8+ T cells and NK cells. Cell Transplant. 2011;20(10):1547–1559. 10.3727/096368910X564076 21396164

[20] Podestà MA, Remuzzi G, Casiraghi F. Mesenchymal stromal cells for transplant tolerance. Front Immunol. 2019;10:1287. 10.3389/fimmu.2019.01287 31231393 PMC6559333

[21] Lan Y-W, Choo K-B, Chen C-M, et al. Hypoxia-preconditioned mesenchymal stem cells attenuate bleomycin-induced pulmonary fibrosis. Stem Cell Res Ther. 2015;6(1):97. 10.1186/s13287-015-0081-6 25986930 PMC4487587

[22] Song Y, Dou H, Li X, et al. Exosomal miR-146a contributes to the enhanced therapeutic efficacy of interleukin-1β-primed mesenchymal stem cells against sepsis. Stem Cells. 2017;35(5):1208–1221. 10.1002/stem.2564 28090688

[23] Miceli V . Use of priming strategies to advance the clinical application of mesenchymal stromal/stem cell-based therapy. World J Stem Cells. 2024;16(1):7–18. 10.4252/wjsc.v16.i1.7 38292438 PMC10824041

[24] Cheng P-P, Liu X-C, Ma P-F, et al. iPSC-MSCs combined with low-dose rapamycin induced islet allograft tolerance through suppressing Th1 and enhancing regulatory T-cell differentiation. Stem Cells Dev. 2015;24(15):1793–1804. 10.1089/scd.2014.0488 25867817 PMC4507355

[25] Ge W, Jiang J, Baroja ML, et al. Infusion of mesenchymal stem cells and rapamycin synergize to attenuate alloimmune responses and promote cardiac allograft tolerance. Am J Transplant. 2009;9(8):1760–1772. 10.1111/j.1600-6143.2009.02721.x 19563344

[26] Sokol CL, Luster AD. The chemokine system in innate immunity. Cold Spring Harb Perspect Biol. 2015;7(5):a016303. 10.1101/cshperspect.a016303 25635046 PMC4448619

[27] Shi Y, Jiao C, Lu X, Nie Y, Li X, Han D. Rapamycin nanoparticles improves drug bioavailability in PLAM treatment by interstitial injection. Orphanet J Rare Dis. 2022;17(1):349. 10.1186/s13023-022-02511-6 36085075 PMC9463820

[28] Haeri A, Osouli M, Bayat F, Alavi S, Dadashzadeh S. Nanomedicine approaches for sirolimus delivery: a review of pharmaceutical properties and preclinical studies. Artif Cells Nanomed Biotechnol. 2018;46(sup1):1–14. 10.1080/21691401.2017.1408123 29186990

[29] Nguyen TT, Pham D-V, Park J, et al. Engineering of hybrid spheroids of mesenchymal stem cells and drug depots for immunomodulating effect in islet xenotransplantation. Sci Adv. 2022;8(34):eabn8614. 10.1126/sciadv.abn8614 36001671 PMC9401619

[30] Xu H, Wang B, Ono M, et al. Targeted disruption of HLA genes via CRISPR-Cas9 generates iPSCs with enhanced immune compatibility. Cell Stem Cell. 2019;24(4):566–578.e7. 10.1016/j.stem.2019.02.005 30853558

