# Supplementary figures and images for: Combined rapamycin and mesenchymal stem/stromal cells derived from induced pluripotent stem cells-mediated delivery of ACVR2B-Fc fusion protein reduces heterotopic ossification in a mouse model of fibrodysplasia ossificans progressiva
Source: JBMR Plus. 2025 Apr 21;9(6):ziaf068. doi: 10.1093/jbmrpl/ziaf068 (PMC12103895; doi:10.1093/jbmrpl/ziaf068)

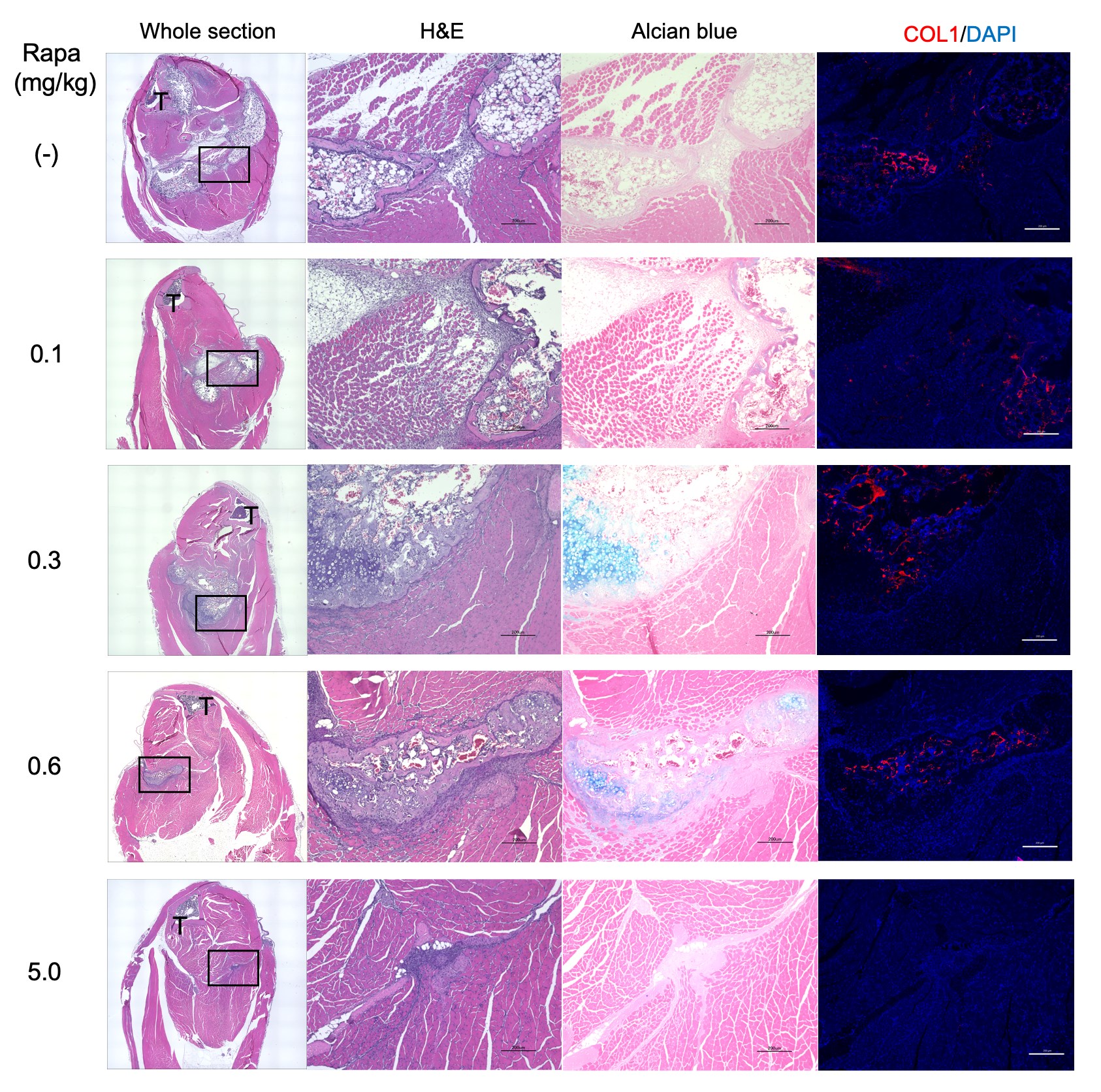

Supplement: Figure_S1_ziaf068 [file figure_s1_ziaf068.jpeg]

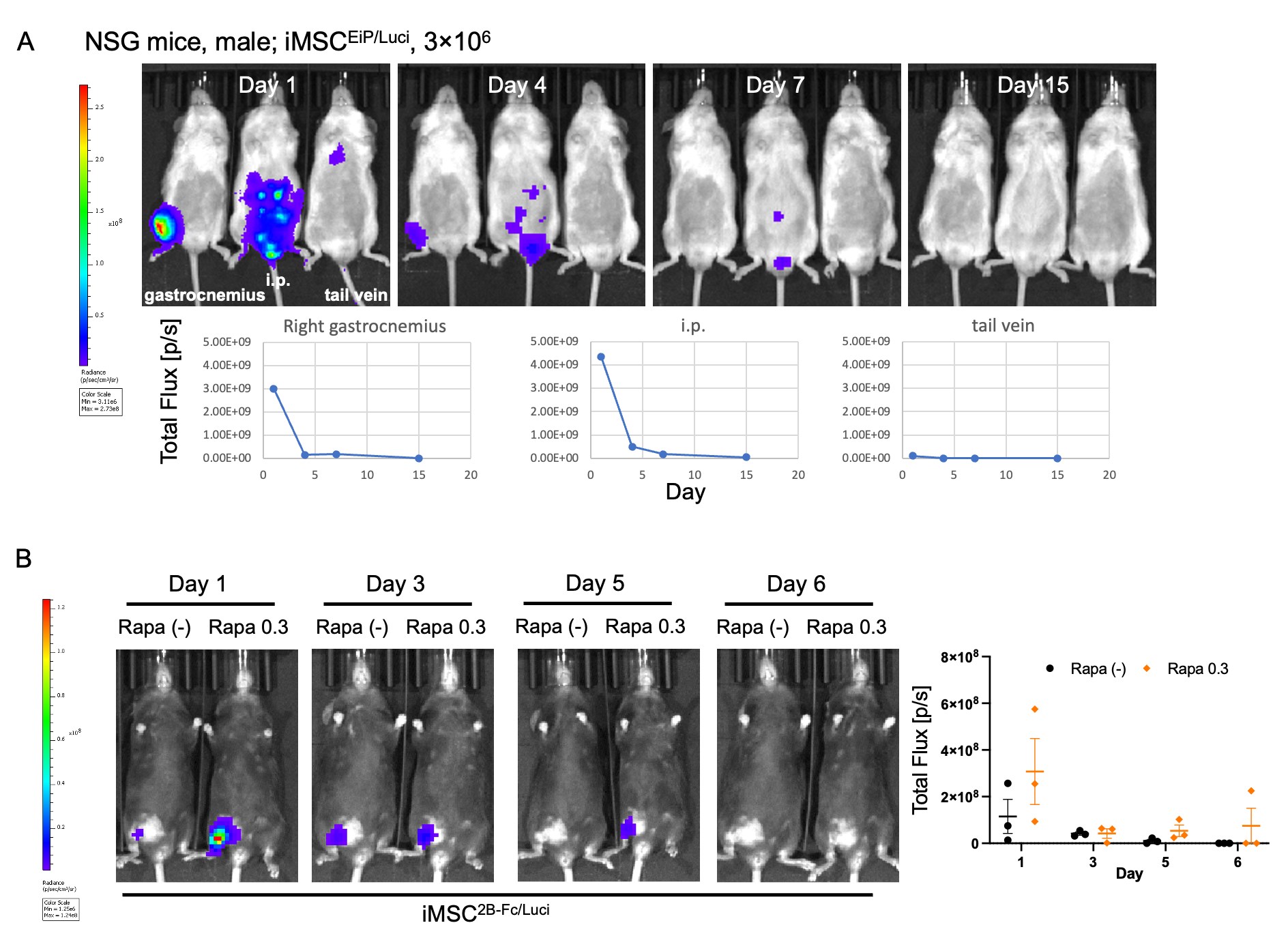

Supplement: Figure_S2_ziaf068 [file figure_s2_ziaf068.jpeg]

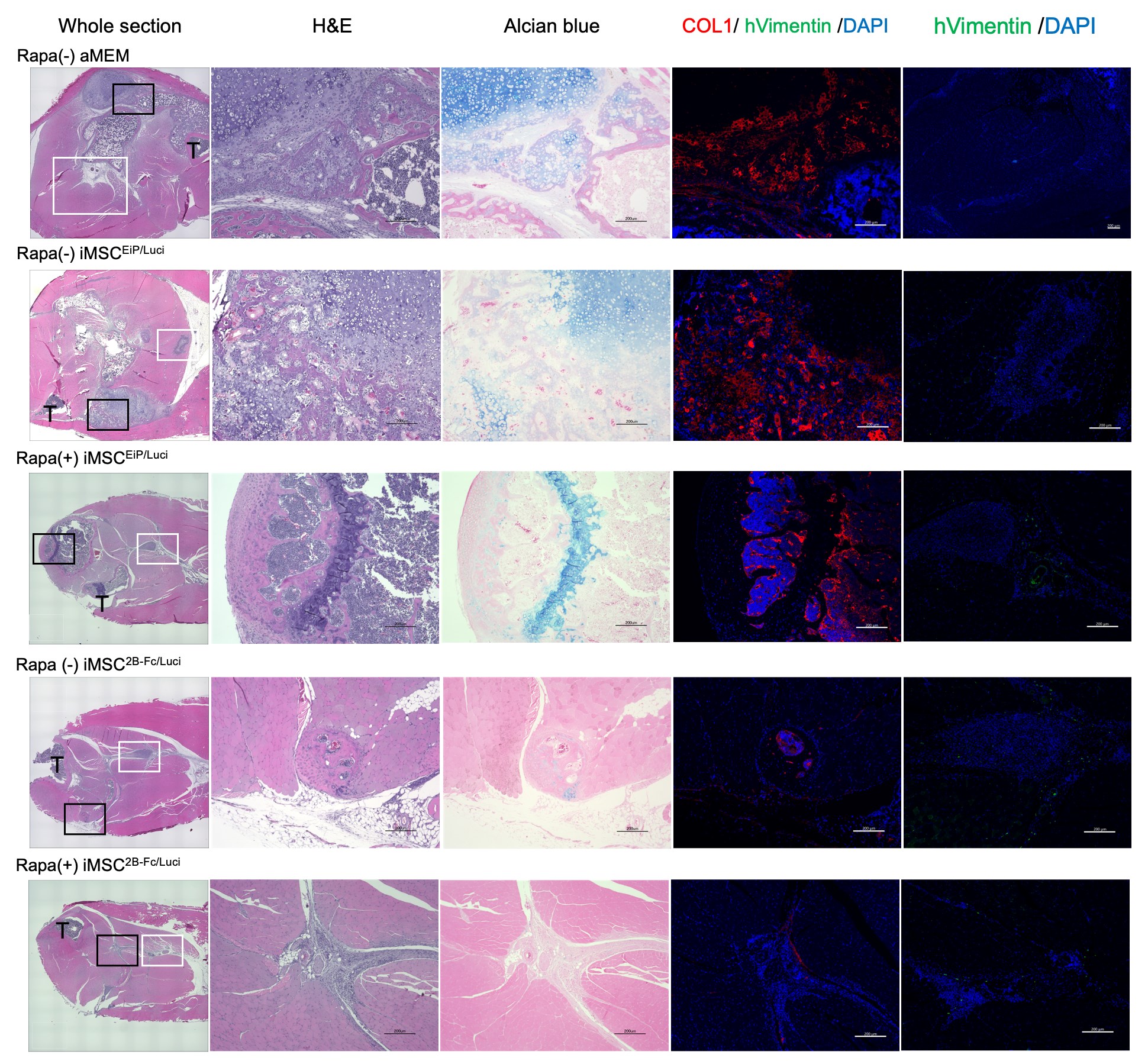

Supplement: Figure_S3_ziaf068 [file figure_s3_ziaf068.jpeg]

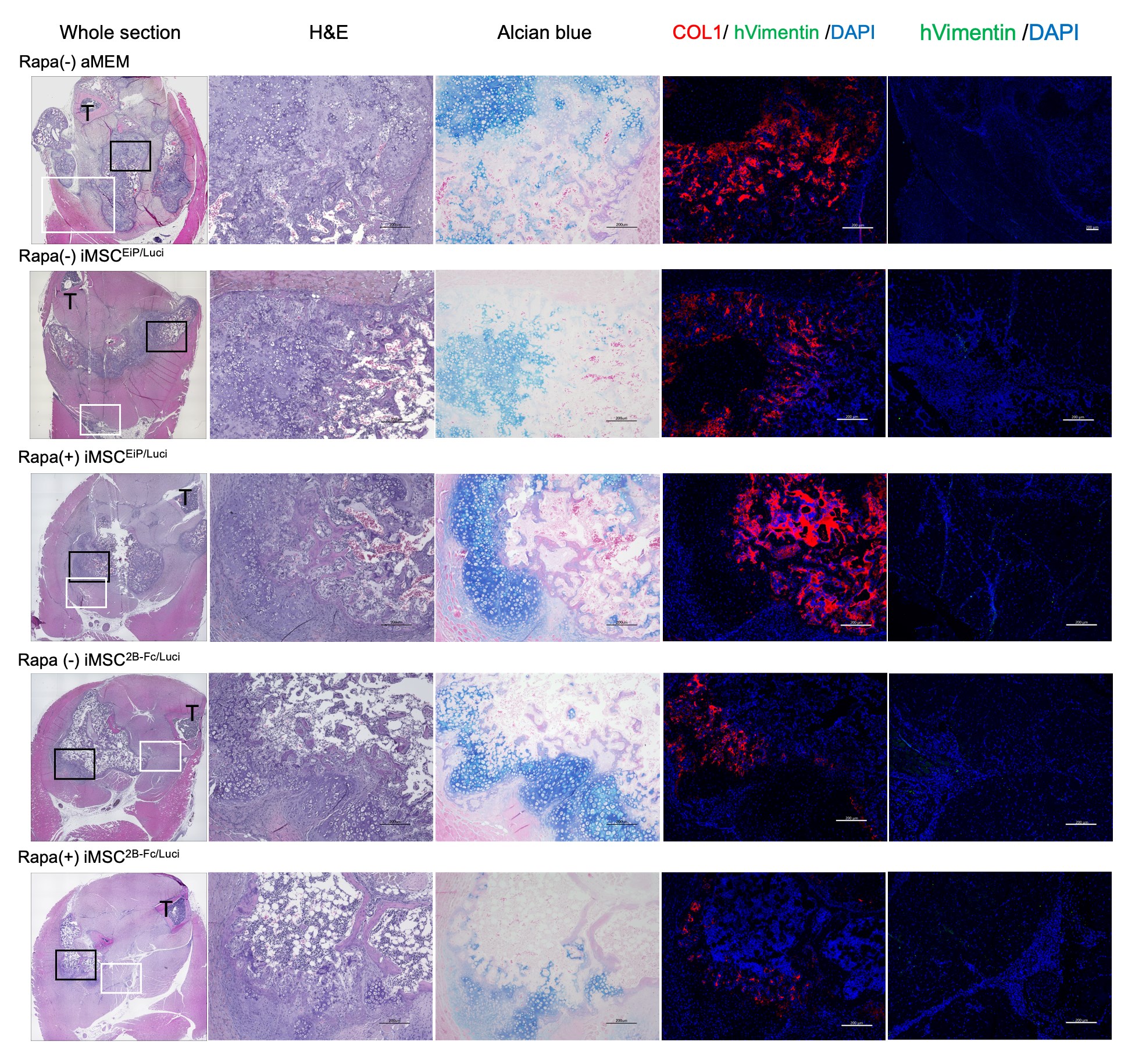

Supplement: Figure_S4_ziaf068 [file figure_s4_ziaf068.jpeg]
