# Supplementary material for: Combined rapamycin and mesenchymal stem/stromal cells derived from induced pluripotent stem cells-mediated delivery of ACVR2B-Fc fusion protein reduces heterotopic ossification in a mouse model of fibrodysplasia ossificans progressiva
Source: JBMR Plus. 2025 Apr 21;9(6):ziaf068. doi: 10.1093/jbmrpl/ziaf068 (PMC12103895; doi:10.1093/jbmrpl/ziaf068)
Supplement: Figure_captions_ziaf068 [file figure_captions_ziaf068.docx]

**Fig S1. Histological and immunohistochemical analysis of HO in Rapamycin dose-dependent model.** H&E staining, Alcian blue (cartilage marker), anti-COL1 (bone marker), and DAPI (nucleus maker) staining are shown. T, Tibia. Black frame, the location of H&E, Alcian blue, and COL1/DAPI staining. Scale bar, 200 µm.

**Fig S2. Tracking donor cells using *in-vivo* imaging after transplantation.** (A) *In-vivo* imaging of donor cells (iMSC^EiP/Luci^, 3×10^6^) within 15 d in the NSG mice. For the left mouse, cells were transplanted by local injection into the gastrocnemius muscle. For the middle mouse, cells were transplanted intraperitoneally. For the right mouse, cells were systemically transplanted through the tail vein. The time course of the luciferase signal of the three administrations was shown on the lower panel. (B) *In-vivo* imaging of 1.5×10^6^ cells of iMSC^2B-Fc/Luci^ within 6 d in the FOP mice. Rapamycin (0.3 mg/kg, 5 times/week) and the control were intraperitoneally administered after CTX injected in the gastrocnemius muscle. Luciferase signals were captured on the next day of cell transplantation. The time course of the luciferase signal was shown on the right panel.

**Fig S3.** **Histological and immunohistochemical analysis of HO in primary HO model.** H&E staining, Alcian blue, anti-COL1, hVimentin (iMSCs transplanted), and DAPI staining are shown. T, Tibia. Black frame, the location of H&E, Alcian blue, and COL1/hVimentin/DAPI staining; white frame, hVimentin/DAPI staining. Scale bar, 200 µm.

**Fig S4. Histological and immunohistochemical analysis of HO in recurrent HO model.** H&E staining, Alcian blue, anti-COL1, hVimentin, and DAPI staining are shown. T, Tibia. Black frame, the location of H&E, Alcian blue, and COL1/hVimentin/DAPI staining; white frame, hVimentin/DAPI staining. Scale bar, 200 µm.
